# Transcranial Direct Current Stimulation of the Medial Prefrontal Cortex Affects Judgments of Moral Violations

**DOI:** 10.3389/fpsyg.2017.01812

**Published:** 2017-10-26

**Authors:** Hong Yuan, Serik Tabarak, Wenxin Su, Yong Liu, Jing Yu, Xu Lei

**Affiliations:** ^1^Sleep and NeuroImaging Center, Faculty of Psychology, Southwest University, Chongqing, China; ^2^Key Laboratory of Cognition and Personality of Ministry of Education, Chongqing, China; ^3^Peking-Tsinghua Center for Life Sciences, Academy for Advanced Interdisciplinary Studies, Peking University, Beijing, China

**Keywords:** moral violations, brain stimulation, mPFC, moral judgments, tDCS

## Abstract

Previous studies show that neural activities in the medial prefrontal cortex (mPFC) are correlated with moral processing during picture viewing tasks. In this study, we applied transcranial direct current stimulation (tDCS) to determine whether this non-invasive brain stimulation technique could modulate the evaluation of moral violations. Sixty-four subjects were randomly recruited, separated into different groups and tested with 42 pairs of pictures depicting moral violations. Each subject was required to rate the pictures two separate times, i.e., before and after tDCS intervention. We found that anodal tDCS (atDCS) increases cortical excitability over the mPFC (between the Fpz and Fp1 positions) as well as the sense of morality and emotional arousal of the subjects. In conclusion, this study indicated that the mPFC plays an important role in moral judgments while modulating ratings of moral violations under tDCS intervention conditions.

## Introduction

Morality is a principle of social behaviors commonly recognized and followed by the majority of society (Graham et al., 2011). Attention to morality has increased as the number of people disobeying this rule has increased. For example, refusal to help elders when they fall has become bizarrely commonplace among Chinese citizens. People have chosen to avoid elders who are falling instead of trying to help them to stand up. Moreover, this behavior is contrary to the rule of morality and thus calls for increased attention and focus on moral processing.

Most recent studies related to morality have focused on correlations between the modulation of the temporal-parietal junction (TPJ) cortex and belief attributions in moral judgments. However, different types of moral judgments, including those related to responsibility, wrongness, belief and blame, recruit distinct brain areas (Guglielmo, 2015). For example, modulating the activation of the right TPJ affects moral judgments when participants read moral scenarios that present conflicting information about the outcome of an action and the intention of the actor (Young et al., 2007; Sellaro et al., 2015; Ye et al., 2015). Moreover, the activity of the medial prefrontal cortex (mPFC) cortex, another area of the brain, strongly correlates with the judgment of images depicting moral violations (Harenski and Hamann, 2006; Decety et al., 2012; Fumagalli and Priori, 2012). Several studies suggest that the mPFC is recruited in general evaluative judgments (Zysset et al., 2002) and integrates emotion into decision making, thereby contributing to moral sensitivity (Damasio, 1994/2015; Decety et al., 2012). Furthermore, solid evidence supports the notion of the recruitment of the mPFC in the process of moral judgment (Greene et al., 2001; Greene and Haidt, 2002; Decety et al., 2012; Fumagalli and Priori, 2012). However, whether the activation of the mPFC affects moral judgment has not been directly confirmed by these studies, and none of these studies have focused on causal relationships.

Transcranial direct current stimulation (tDCS) is a tool that applies a micro-electric current in the brain capable of modulating cognitive brain functions. Specifically, the magnitude of the micro-current is controlled within 1–2 mA of a value suitable for human beings. The application of an anodal tDCS (atDCS) stimulates greater higher excitability in the target cortex, whereas a tDCS has an effect similar to that of a placebo (Nitsche and Paulus, 2000). Several studies demonstrate that tDCS affects cognitive processes, including motion control (Nitsche et al., 2008), executive function and verbal ability (Boggio et al., 2011).

However, research regarding the application of tDCS interventions during the performance of moral image tasks, especially those addressing real-life moral issues, remains extremely limited. Thus, this study will examine whether the application of the non-invasive brain stimulation technique of tDCS is able to modulate the evaluation of moral violations.

## Methods

### Subjects

A total of 64 volunteers were recruited from Southwest University and randomly divided into two groups, i.e., an atDCS group and a sham tDCS group. Twenty-six of the subjects were female, and 38 were male (mean age: 23.57 years old with a standard deviation of 2.1). No subject histories contained major psychological disorders or vision deficiencies, nor were any subjects taking substances or medications that could potentially have affected their mental concentration. Before beginning the experiment, all subjects read the instructions and were permitted to ask questions about tDCS and the experimental safety guarantee. This study was approved by the ethics committee of the faculty of Psychology at Southwest University of China.

### Materials

This study involved improvements that were developed based on the moral and immoral picture stimuli patterns used by Harenski and Hamann (2006). These improvements included the addition of pictures that were closely representative of the current existing moral problems in China. Consequently, 76 pairs of pictures were selected from the International Affective Picture System (IAPS) and other popular media platforms. Each pair of pictures consisted of one depicting a normal behavior in life and another depicting an immoral behavior occurring in the same social content.

**FIGURE 1:**
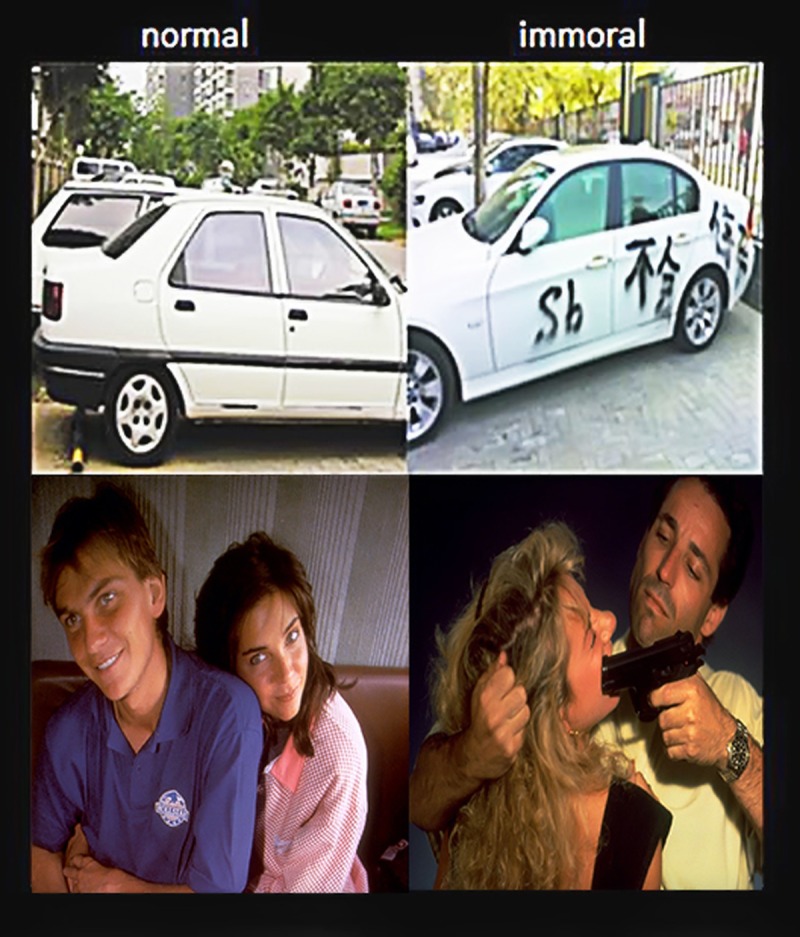
Moral and immoral picture stimuli. The first row shows the type of pictures obtained from the internet, and the second row shows the type of pictures obtained from the International Affective Picture System (IAPS, Nos. 4700 and 6560).

For this experiment, a pilot study was preliminarily conducted on 20 subjects with the aim of filtering the pictures to create a standardized norm. The subjects were asked to separately rate the degrees of moral violation, emotional arousal and content complexity of each picture on several 7-point Likert scales. Here, for moral violation, “1” indicated very little moral violation, and “7” indicated serious moral violation. For the emotional arousal scale, “1” indicated very unattractive, and “7” indicated very attractive. For the complexity of the picture’s content scale, “1” indicated very non-complex, and “7” indicated very complex. This pilot study resulted in the selection of 42 pairs (75 of which were chosen from the popular media platforms, and the rest of 9 pictures were chosen from the IAPS) of pictures from a total of 76 pairs. **Figure 1** provides examples of the pairs of pictures.

**FIGURE 2:**
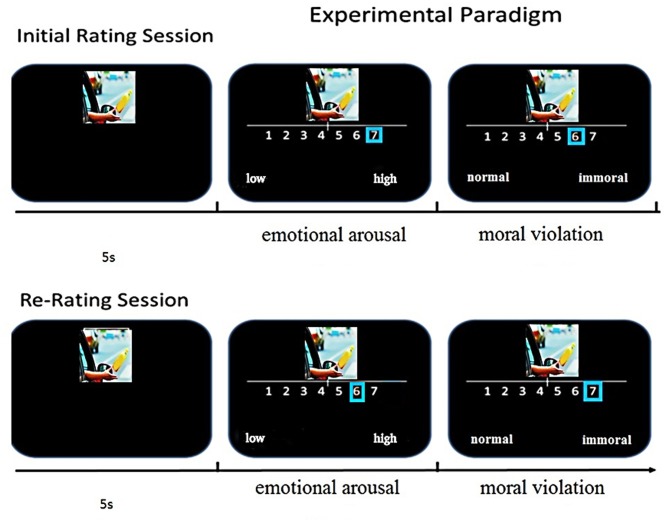
Experimental task design. During the initial rating session, the subjects viewed moral and immoral pictures. In the re-rating session, the subjects rated the same pictures again without being reminded of their prior ratings.

After the pilot study, the development of the formal experimental procedures was continued. These procedures included a preliminary test and a formal test. The preliminary test was designed to inform the subjects of the requirements of the evaluations. The formal test comprised an initial rating session and a re-rating session, and tDCS was conducted in the time between these sessions. The subjects were asked to rate the pictures in the first session and then re-rate the pictures in the second session. The subjects were not expecting the re-rating test. In these two sessions, each pair of pictures appeared for 5 s with an interstimulus interval of 2 s, which left no time for evaluation. Each round of the session (i.e., the initial rating session and the re-rating session) lasted approximately 30 min. The picture presentation sequence differed between the two sessions, and the process of the re-rating session is detailed in **Figure 2**.

### tDCS Parameters

**FIGURE 3:**
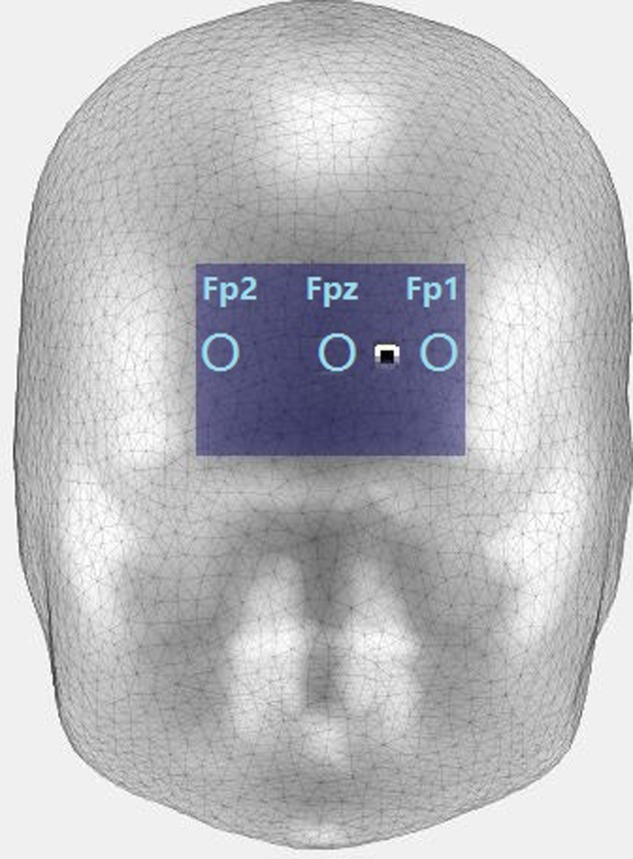
Target location of the transcranial direct current stimulation (tDCS) electrode. The anode electrodes were placed in the Fpz-Fp1 site, and the extra-encephalic reference was placed over the right shoulder (Civai et al., 2015; Nakamura and Kawabata, 2015).

By using a DC-STIMULATOR PLUS (NeuroConn, Germany), a constant 1.5-mA current flow was applied via a pair of a saline-soaked sponge electrodes (35 cm^2^; current density: 0.057 mA/cm^2^). As shown in **Figure 3**, the electrodes were placed in the centers of Fp1 and Fp2 based on the EEG 10-20 system. On the basis of previous studies, the ‘reference’ electrode was fixed extra-cephalically on the right arm to avoid any ‘reference’ electrode interference with the mPFC cortex (Civai et al., 2015; Nakamura and Kawabata, 2015).

### Statistical Analysis

In this study, the ratings were analyzed by 3-way ANOVA using time (initial rating vs. re-rating) and image type (immoral vs. normal) as within-subject factors and treatment (tDCS: anode vs. sham) as a between-subject factor. We conducted two 3-way ANOVAs, i.e., one for moral violation and one for emotional arousal.

## Results

**FIGURE 4:**
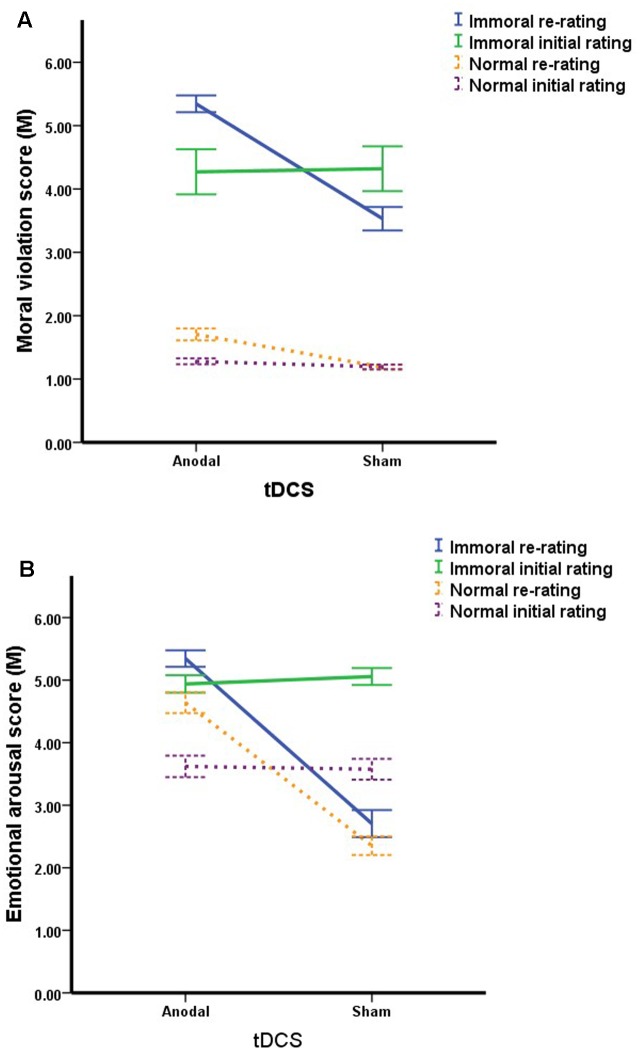
**(A)** Changes in the moral violation ratings by image type and treatment group. tDCS enhanced the ratings for both the immoral pictures and normal pictures. **(B)** Changes in the emotional arousal ratings by image type and treatment group. tDCS enhanced the ratings for both the immoral and the normal pictures. An acute decline in the re-rating scores of the images occurred due to the use of the same stimuli. The error bars indicate ±1 SE.

The primary effect of image type on moral violation rating was significant (**Figure 4A**). The immoral images evoked a greater moral sense than the normal images in general [*F*(1,62) = 376.50, *p* < 0.001]. Moreover, a noticeable interaction was observed between treatment and time [*F*(1,62) = 15.028, *p* < 0.001]. Specifically, the results of the re-rating scores indicated that the ratings of the anodal group (3.52 ± 0.86) were significantly higher than those of the sham group [2.36 ± 0.89; *t*(1,62) = 1.165*, p* < 0.001]. Additionally, no difference was observed between the anodal and sham groups in the initial rating session [*t*(1,62) = 0.19*, p* = 0.940], indicating that the baseline moral sense of the two groups in the initial ratings were not significantly different (**Figure 1**). Notably, no other effects were found.

In the emotional arousal ratings (**Figure 4B**), the primary effect of the image type was significant [*F*(1,62) = 62.987*, p* < 0.001], indicating the immoral images evoked greater emotional arousal than the normal images. Another primary significant effect was that of time [*F*(1,62) = 23.957*, p* < 0.001]. These data indicated the re-rating score was lower than the initial rating score, demonstrating the trend of an overall decline in emotional arousal.

Furthermore, a strong connection between treatment and time was observed [*F*(1,62) = 15.028*, p* < 0.001]. The rating scores of the anodal group in the re-rating session (4.99 ± 0.12) were significantly higher than the initial ratings (4.27 ± 0.11). However, the opposite trend was observed in the sham group [*t*(1,62) = 2.463*, p* < 0.001], i.e., the re-rating scores of the sham group (2.53 ± 0.09) were lower than the initial ratings (4.31 ± 0.12). This finding indicated an acute practice effect in the sham group. Notably, no difference was observed between the anodal group and the sham group in the initial ratings [*t*(1,62) = 0.37*, p* = 0.818; **Figure 1**]. No other effects were significant.

## Discussion

This study aimed to use tDCS to modulate the judgment of moral violation. We concluded that the anodic stimulus over the mPFC induced a greater sense of morality in this study. Specifically, upon the use of atDCS over the mPFC, the subjects tended to rate the severity of the moral violations in the pictures higher than the sham group. In comparison, the re-rating scores were relatively lower than the initial rating scores in the sham stimulation group. Consequently, both findings indicated that the activation of the mPFC produced a noticeable effect on the sense of morality of the subjects.

Theoretically, presenting the same pictures to the subjects during both rating sessions would influence their judgments and cause decreases in the sense of moral violation and emotional arousal in the re-rating session of sham group. Emotion plays an important role in the sense of morality, and moral judgments generally necessitate emotional involvement (Greene et al., 2001; Harenski and Hamann, 2006; Decety et al., 2012). We believe that the regression effect in the sham group was related to a decrease in the level of emotional arousal. However, the study results revealed a significant increase in both ratings (i.e., moral violation and emotional arousal) in the anodal group (**Figure 4**).

Additionally, the repetition of the same pictures before and after the tDCS intervention might have strengthened the subjects’ memories, which would have further affected the re-rating scores. However, the complexity of our task greatly undermined this potential influential impact. First, 84 pictures (*n* = 84) were chosen for this experiment, Second, each picture had two rating procedures (emotional arousal and moral violation severity), both of which were rated on a Likert scale that ranged from “1” to “7.” Third, the pictures were randomly presented during the re-rating session. Fourth, the subjects waited for a time period of 30 min between the initial and re-rating sessions, and the subjects were not expecting the re-rating session. Therefore, all these four aspects together made it difficult for participants to remember their initial rating choices. Consequently, the re-rating scores most likely accurately reflected the subjects’ attitudes regardless of their memories.

However, this study has several limitations. First, different moral judgment tasks require different types of equipment and the integration of various information from different sources (Guglielmo, 2015). These differences indicate that our results may be limited to tasks involving judgments of moral violations in images. Second, the accuracy of tDCS is rather low and might lead to stimulus generalization that causes activation of the entire prefrontal cortex. Third, the current pathways in the medial cortex remain unclear (Yuan et al., 2015), and the individual differences in cerebral structures may also affect these pathways. Thus, additional effort is needed in this area of study. We believe that future studies should try to verify the effectiveness of the stimulation of the mPFC. Moreover, individual differences should be considered by combining imaging data to customize and optimize the stimulus parameters (Yuan et al., 2015).

## Conclusion

This study indicated that the mPFC plays an important role in moral judgments. Anodic tDCS indeed has enhanced excitability in the mPFC, which increases subjects’ sense of morality and emotional arousal. Furthermore, in this study, we updated an old paradigm to create a new paradigm that more closely resembles current existing moral problems.

## Ethics Statement

Written informed consent was obtained after detailed explanation of the study protocol, which was approved by the Ethics Committee of Southwest University. All procedures were conducted in accordance with the sixth revision of the Declaration of Helsinki.

## Author Contributions

HY, contributed as guarantor of integrity of entire study and made study concept. ST contributed to the study design, literature research, manuscript preparation and revision. WS contributed to the statistical analysis and manuscript editing. YL contributed to the data acquisition and data interpretation. JY contributed to the manuscript definition of intellectual content and manuscript editing. XL contributed to the manuscript final version approval and also contributed as guarantor of integrity of entire study.

## Conflict of Interest Statement

The authors declare that the research was conducted in the absence of any commercial or financial relationships that could be construed as a potential conflict of interest.
